# Gradedness of gist perception with scenes depends on concurrent task load: awareness under high load is more graded due to noise suppression

**DOI:** 10.1093/nc/niag054

**Published:** 2026-08-24

**Authors:** Suraj Kumar, Narayanan Srinivasan

**Affiliations:** Department of Cognitive Science, Indian Institute of Technology Kanpur, Kanpur, Uttar Pradesh 208016, India; Department of Cognitive Science, Indian Institute of Technology Kanpur, Kanpur, Uttar Pradesh 208016, India

**Keywords:** visual awareness, gradedness, attention, perceptual load, scene gist

## Abstract

There is growing evidence that the visual awareness of objects is graded. However, it is unclear whether scene perception is graded and whether attention modulates that gradedness. We addressed these questions in two experiments in which participants identified scenes as indoor or outdoor. Attention was manipulated using a concurrent perceptual load (low or high) task. Scene contrast (Experiment 1) and noise added to scenes (Experiment 2) were manipulated in the study. Participants made a four-point perceptual awareness scale (PAS) rating following the scene judgment. The data on the accuracy of the scene judgments and the mean PAS ratings as a function of contrast (Experiment 1) or noise (Experiment 2) were fitted with a four-parameter logistic function. We predicted lesser PAS rating function slopes in the high-load condition, indicating more graded awareness. Scene perception was better (lower threshold) under low load across experiments indicating more attentional resources benefit scene processing. Crucially, PAS function slopes were shallower under high load in both experiments, indicating that awareness becomes more graded when attentional resources are limited. In Experiment 2, accuracy at high noise levels (the lower asymptote) was higher in the high load condition suggesting that high load suppresses external noise during scene perception. The difference in the lower asymptote for accuracy between load conditions correlated negatively with the difference in PAS ratings slope linking noise suppression to the gradedness effect. Together, these findings support a graded account of visual awareness and demonstrate that attention modulates scene perception clarity by suppressing external noise.

## Introduction

Whether consciousness is discrete or graded and whether its all-or-none/graded nature is dependent on the phenomena under consideration has been of interest to consciousness science (Cleeremans et al. 2020, Dehaene and Changeux 2011, Mashour et al. 2020, Windey et al. 2014, Windey and Cleeremans 2015). The Global Neuronal Workspace Theory (GNWT) treats awareness as a discrete phenomenon with only two states, full awareness or no awareness (Sergent and Dehaene 2004, Vugt et al. 2018), with a sharp and almost discontinuous transition once stimulus activation crosses threshold. In contrast, the Recurrent Processing Theory (Lamme 2010) and the Radical Plasticity Hypothesis (Cleeremans et al. 2020) treat awareness as continuous and graded. Empirical support exists on both sides, with some studies reporting a discrete transition into awareness (Del Cul et al. 2007, Sekar et al. 2013, Asplund et al. 2014) and others finding graded visual experience across diverse stimuli and tasks (Sandberg et al. 2010, Fazekas and Overgaard 2016, 2018, Overgaard 2017, Derda et al. 2019, Jimenez et al. 2020, 2021, Thiruvasagam and Srinivasan 2021).

This mixed evidence has prompted attempts to reconcile discrete and graded accounts rather than treat them as strictly opposed. The partial awareness hypothesis (Kouider et al. 2010), developed within the GNWT framework, posits that conscious perception does not necessarily involve the holistic, all-or-none ignition of a complete stimulus representation. Instead, different features or levels of a stimulus, such as low-level visual properties (e.g. color, orientation), intermediate features (e.g. shape), or higher-level semantic attributes (e.g. word meaning), can independently gain access to consciousness. What appears as graded awareness at the subjective level may therefore reflect partial access to some components of a hierarchical representation while the observer remains unaware of others.

A different approach is offered by the levels of processing (LoP) hypothesis (Windey et al. 2014, Windey and Cleeremans 2015, Jimenez et al. 2020, Filimonov et al. 2024), which locates the source of gradedness in the task rather than in the representation. LoP argues that the level of processing required by a task shapes gradedness, with more gradedness under low-level tasks. When identifying masked stimuli, for instance, awareness was more graded under color discrimination than under number judgment (Windey et al. 2013, Anzulewicz et al. 2015, Jimenez et al. 2021). On this view, gradedness is determined largely by the representational level a task recruits, with little role accorded to other cognitive factors. Studies based on these approaches typically use the perceptual awareness scale (PAS) scale (Sandberg and Overgaard 2015, Overgaard and Sandberg 2021), which a four-point scale to measure levels of subjective clarity or visibility. The steepness parameter of the function that is fitted for mean PAS ratings is used as the measure of gradedness.

Both the partial awareness and LoP accounts leave underspecified how additional cognitive factors, most notably attention, interact with task demands to shape the graded or dichotomous character of awareness. The multi-factor model proposed by Fazekas and Overgaard (2018) offers a more integrative framework, treating conscious experience as shaped by the interaction of stimulus strength, task demands, and attention. On this account, attention does not merely gate whether a representation reaches consciousness; it modulates the quality, stability, and precision of representations across LoP.

Within this framework, one especially relevant aspect is attentional resources available for a task, which depends in turn on the attentional demands of a concurrent task. Previous studies have shown that when participants were asked to perform two tasks, performance on the secondary task decreases when more attentional resources are given to the primary task (Kahneman 1973, Pashler 1994, Wickens 1980). How such attentional resource allocation influences visual awareness is not fully understood. Fazekas and Overgaard (2018) describe residual attention spilling over to stimuli outside the focus of attention, which are then represented by low-quality population codes and experienced only faintly. Scope of attention matters too: Thiruvasagam and Srinivasan (2021) showed, using a letter discrimination task, that a broad scope of attention produced more graded awareness than a narrow one. Together, these findings converge on the prediction that conditions linked to less attentional resources should yield more graded, rather than more dichotomous, conscious experience.

If attention shapes the gradedness of awareness, it should do so through known attentional mechanisms that affect visual representation. Multiple paradigms have shown that attention influences visual appearance (Carrasco et al. 2004, Liu et al. 2009, Carrasco 2011, Carrasco and Barbot 2019, Mishra and Srinivasan 2017). Exogenous spatial attention increases the perceived contrast of Gabor patches at cued compared to uncued locations (Ling et al. 2004), and endogenous spatial attention produces similar effects (Liu et al. 2009). These effects are typically explained by two mechanisms: signal enhancement and external noise suppression (Carrasco 2011).

Beyond these local effects of spatial attention, visual awareness is also shaped by the broader availability of attentional resources. A central manipulation of this availability is processing load (Lavie 1995, Murphy et al. 2016). The perceptual load framework treats attention as a capacity-limited system in which processing depends on the resources required for a task (Lavie 1995). Lavie et al. (2014) extended this to visual awareness, arguing that high load exhausts capacity while low load leaves spare capacity that supports full awareness of both relevant and irrelevant information. Using a dual task that measured the contrast response function of a concurrent orientation discrimination under different load conditions, they showed that high load shifted the function rightward, indicating that load modulated the contrast required to reach above-threshold accuracy. A key limitation is that this study measured objective performance only and did not include a subjective measure of perceptual clarity, leaving open the question of how load affects the gradedness of awareness itself.

Gist perception is a particularly informative test case because the relationship between attention and gist itself remains contested. Some researchers have argued that gist perception does not require attention (Koch and Tsuchiya 2007, Li et al. 2002, VanRullen et al. 2005), while others hold that it does (Evans and Treisman 2004, Mack and Clarke 2012, Brand and Johnson 2018). This debate has been pursued largely through dual task studies asking whether a concurrent task disrupts gist performance. Crucially, these studies have treated gist perception as an all-or-none outcome and have not asked whether scene gist perception is itself graded, nor whether its gradedness varies with the availability of attentional resources.

We addressed this gap using a dual task paradigm combining a letter identification task with a scene gist perception task (indoor versus outdoor categorization). Attention was manipulated through the perceptual load of the letter identification task, with higher load demanding more resources. Participants were instructed to prioritize letter identification so that attentional resources were appropriately allocated to the primary task across load conditions. We measured accuracy for both tasks. We also obtained perceptual awareness ratings (PAS ratings) for scene clarity or visibility. Later, we fitted a logistic function relating stimulus quality (contrast/noise level) to mean subjective awareness ratings obtained using the PAS scale as well as for scene identification accuracy. Given that a concurrent task reduces attentional resources available for a secondary task (Lavie 1995, Fazekas and Overgaard 2016, 2018), we hypothesized that scene gist perception under high load would be more graded than under low load, reflected in a shallower slope of the PAS rating function.

We report two experiments. Experiment 1 manipulated scene contrast and obtained psychometric curves for objective accuracy and subjective awareness under high and low perceptual load. Experiment 2 manipulated external additive noise (Lu and Dosher 1998) rather than contrast, allowing us to identify the attentional mechanism (signal enhancement versus external noise suppression) underlying any load-related change in gradedness.

## Experiment 1

In Experiment 1, participants categorized scenes as indoor or outdoor while performing a concurrent letter identification task, with scene contrast varied to obtain full psychometric functions. Load was manipulated through distractor heterogeneity, with identical distractors in the low load condition and different distractors in the high load condition. On each trial, participants identified the target letter, categorized the scene, and then rated awareness on the PAS (Sandberg and Overgaard 2015, Anzulewicz et al. 2015). We expected letter identification to be better under low than high load, consistent with prior work (Lavie 1995, Murphy et al. 2016), and scene identification to show a lower threshold under low load given the greater processing resources available for the concurrent task. For gradedness, earlier findings that easier, less resource-demanding conditions yield more graded awareness, whether through low-level tasks (Windey et al. 2014) or global over local processing (Thiruvasagam and Srinivasan 2021), led us to predict shallower PAS slopes under high load, where fewer resources are available for scene processing.

### Method

#### Participants

Thirty-three naïve volunteers (*M*_age_ = 24.21 years, *SD_age_* = 4.01 years, females = 11) with normal or corrected-to-normal vision participated in the current study after providing informed consent. They were all compensated with Rs. 100/−. The sample size was estimated using G*Power 3.1 (Erdfelder et al. 1996, Faul et al. 2007) with α = 0.05, power = 0.85, and the effect-size, *d* = 0.56 for difference in slope for processing levels from Windey et al. (2013). The estimated sample size was 25. Data from eight participants were removed because of bad fit (*R^2^* < 0.8). The study was approved by the Institutional Ethics Committee.

#### Stimuli and apparatus

The stimuli were real-world scenes presented on a 24-inch monitor with refresh rate 100 Hz with screen-resolution at 1920 × 1080 pixels and RGB color space. These scene images were taken from the ObScene database (Andrade et al. 2024) and CB Database (Sareen et al. 2016) and divided into indoor and outdoor categories. In the indoor category, out of 314 total images, the images with extreme dark or light shades were removed and finally 257 images were selected to be used in the experiment. Similarly, in the outdoor category, out of 284 total images, 240 images were selected to be used in the experiment. The size of the images was 650 pixels × 500 pixels. After conversion to a grayscale image, the contrast of all these images was normalized using a histogram equalization method implemented in python. Finally, the contrast of these greyscale images was further reduced to eight levels (3%, 6%, 9%, 12%, 15%, 18%, 21%, 24%) to be used in the current experiment. The mean luminance of all the scenes was kept the same. All the programs that were used for preprocessing and stimuli preparation are available at OSF. Each image subtended a visual angle of 17.06^°^ × 15.18°. The stimuli were presented on a grey background (58 cd/m^2^). The experiment took place in a dark room, and participants viewed the display from 60 cm. Stimulus presentation and timings were controlled using PsychoPy software (version 2024.1.5) (Peirce et al. 2019). Responses were obtained using the keyboard.

#### Procedure

We used a dual task paradigm. The trial structure for Experiment 1 is shown in Fig. 1. Participants first performed a letter identification task (“X” or “N” letter presented among a set of distractor letters). Then as part of the second task, they first performed scene-identification (whether the scene was an indoor or outdoor scene) and then provided visual clarity ratings for the perceived scenes. The contrast of the scene images presented in the secondary task varied (as explained in the stimuli section above), however, the contrast of the letters presented in the primary task were not manipulated. The images were randomly chosen from the selected dataset, and an image was presented only once in the experiment to minimize any potential memory effects. The clarity rating was obtained using a 4-point PAS (Sandberg and Overgaard 2015). Participants were instructed to prioritize their performance in the letter-identification task. Six letters were presented in a circular array (0.6° × 0.6°, 3.5° between each letter, 5.6° from the center). In the high load condition, “X” or “N” was presented among other letters such as “K”, “F”, “S”, “U”, “P”. In the low load condition “X” or “N” was presented among the multiple ‘O’s (see Fig. 1). All the letters were blue in color presented over a greyscale scene-image in the background.

**Figure 1:**
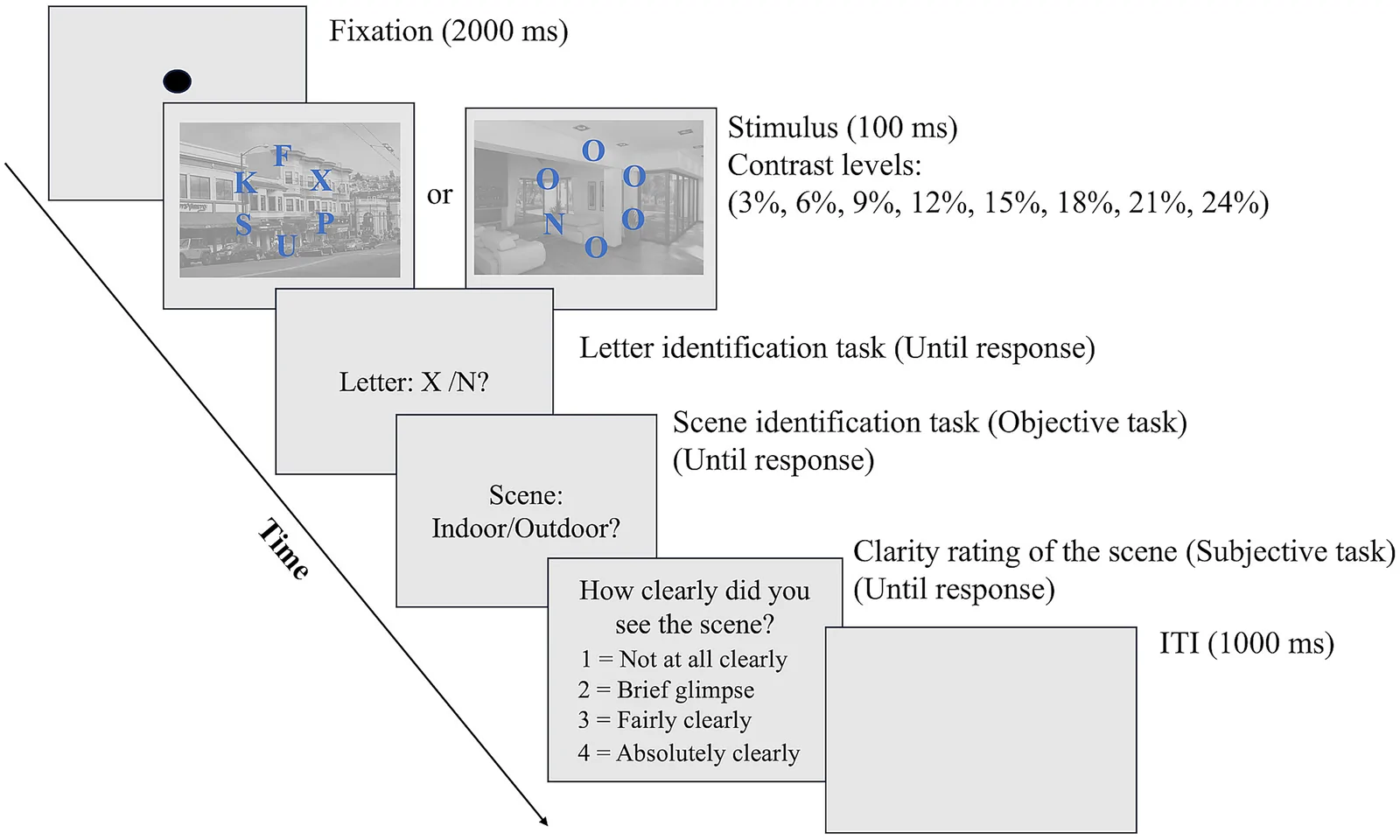
Trial structure of Experiment 1.

Every trial started with a fixation dot at the center of the screen for 2000 ms. This was followed by a stimulus presentation (letter array and scene) for 100 ms. After stimulus presentation, participants first completed the letter identification task and then performed the scene identification task (indoor or outdoor scene). For the letter-identification, participants were instructed to press the key “9” if letter “X” was present among other letters or press the key “0” if “N” was present among the other letters. For the scene-identification, participants were instructed to respond via a mouse-click on the indoor/outdoor options presented on the screen. and then click on the PAS rating scale presented on the screen. They then reported on the visibility of the scene using the 4-point PAS scale presented on the screen using a mouse-click (see Fig. 1). A blank grey screen was presented during the inter-trial interval for 1000 ms.

The PAS scale description, instructions and training were provided as suggested by earlier studies (Anzulewicz et al. 2015, Sandberg and Overgaard 2015, Jimenez et al. 2020, Overgaard and Sandberg 2021). For subjective clarity ratings of the scenes, participants were explicitly instructed to not provide the ratings based on their objective task performance and provide the ratings based only on how clearly they saw the scene. We used the PAS scale with four labels for the visibility ratings (for the question: “How clearly did you see the scene?”): (i) Not at all clearly, (ii) Brief glimpse, (iii) Fairly clearly, and (iv) Absolutely clearly (Anzulewicz et al. 2015, Sandberg and Overgaard 2015, Overgaard and Sandberg 2021). In each trial, the PAS scale ratings along with the labels remained on screen until the clarity rating until response.

Before the main experiment, participants performed separate practice trials for letter-identification (for both high load and low load conditions) as well as for scene-identification (16 trials each). This was followed by a practice session with letters and the scene together (18 trials). Visual feedback for correct/incorrect responses was provided during practice trials. The main experiment had a total of 320 trials. There were two blocks of high load condition and two blocks of low load condition with 80 trials in each block (20 trials per contrast). The order of high load and low load blocks were counterbalanced across participants. The experiment lasted approximately for 45–50 min with a self-paced break after every block.

#### Data analysis

We analyzed participants’ performance in the letter-identification and scene perception tasks separately. For the letter task, participants’ overall accuracy was analyzed to validate the load manipulation. For the scene accuracy and perceptual ratings analysis, only those trials were included in which participants had responded correctly in the letter identification task. For the scene task, a psychometric curve was obtained for each participant’s objective performance (i.e. accuracy) and subjective ratings (mean PAS ratings) as a function of stimulus-contrast in both the load conditions. The parameters from the fits as well as letter identification accuracy as a function of load was compared using either a paired *t-*test or a Wilcoxon signed rank test depending on the normality of the data. Shapiro–Wilk test was performed to check the normality of the data. The data and codes are publicly available at OSF.

For the scene-identification data (for both, objective performance, i.e. scene-identification accuracy and subjective-ratings, i.e. mean PAS-ratings), we fitted a four-parameter nonlinear model (equation 1) for individual subjects based on the procedure (Sandberg et al. 2011, Windey et al. 2013, Thiruvasagam and Srinivasan 2021).

\begin{eqnarray*}f(x)=a+\frac{b-a}{1+{\mathit{\exp}}^{\frac{c-x}{d}}} \end{eqnarray*}

In this four-parameter model, the free parameters “*a*” and “*b*” reflect the lower and the upper boundaries of the psychometric function. “*c*” and “*d*” represent the threshold and steepness of the curves respectively. The steepness parameter of the curve *d* is inversely proportional to slope. A larger *d* value implies a smaller slope and more gradedness (Sandberg et al. 2011, Windey et al. 2013, Thiruvasagam and Srinivasan 2021). A smaller *d* value implies a larger slope and less gradedness. The differences in the threshold parameter “*c*” would indicate capacity limitations in processing as a function of load. While we could fit the data with a two-parameter function (only *c* and *d*) with fixed lower and upper asymptotes, we fitted the data with a four-parameter function since hypotheses in Experiment 2 required fitting with a four-parameter function. The curve fitting was carried out using scipy.optimize library of Python 3.12.3. For all the conditions reported below, the average goodness of fit of the curves, i.e. *R^2^* > 0.80 indicating a good fit of the four-parameter nonlinear model for the data. All statistical tests were done using R software.

We also analyzed the frequency distribution of each of the four PAS rating values obtained for subjective clarity of scenes to see how mean of each of these values changed as a function of contrast and load. We performed a 2-way repeated measures ANOVA for each PAS rating value with contrast and load as the factors. Also, to examine whether the *d* parameter was affected by accuracy differences between conditions, we conducted Spearman’s rank order correlation between the overall accuracy of individual subjects and their *d* parameter from the objective performance and PAS rating curves.

## Results

### Letter identification accuracy

Accuracy for letter identification was significantly different in the high load compared to the low load condition, *t*(24*)* = 8.91, *P* < .01, Cohen’s *d =* 1.78. Letter identification in the high load condition (*M* = 74.75%, *SD* = 8.24%) was indeed more difficult compared to low load (*M* = 87.05%, *SD* = 9.26%) condition showing that load manipulation worked effectively.

### Scene perception results

#### Overall scene identification accuracy

Overall accuracy for scene identification dropped off from the low load (*M* = 79.37%, *SD* = 7.61%) to the high (*M* = 76.90%, *SD* = 8.01) load condition, *t*(24) = 2.85, *P* = .004, *d =* 0.57 indicating that the high load condition restricted the processing of scene information more compared to the low load condition. The overall proportion of correctly identified indoor/outdoor scenes were close for both categories: 80.88% for indoor scenes and 80.92% for outdoor scenes. A paired *t-*test showed no difference in accuracy for indoor and outdoor scenes, *t*(24) = 0.04, *P* = .96, *d =* 0.01.

#### Curve fitting results for scene identification accuracy

The four-parameter psychometric model (equation 1) was fitted to the individual subject’s objective performance, i.e. mean accuracy in identifying indoor/outdoor scenes during high and low perceptual load conditions at different levels of stimulus-contrast. Curve fits for group mean data are shown in Fig. 2. The nonlinear model provided a good fit for the individual subjects’ performance data with group mean *R*^2^ = 0.91 (*SD =* 0.05) for the low load condition and *R*^2^ = 0.90 (*SD =* 0.06) for the high load condition. The results for threshold *c*, steepness parameter *d,* lower asymptote *a*, and upper asymptote *b* for the objective task performance for high and low load conditions are shown in Fig. 3a–d and the mean and SD values are presented in Table 1.

**Figure 2:**
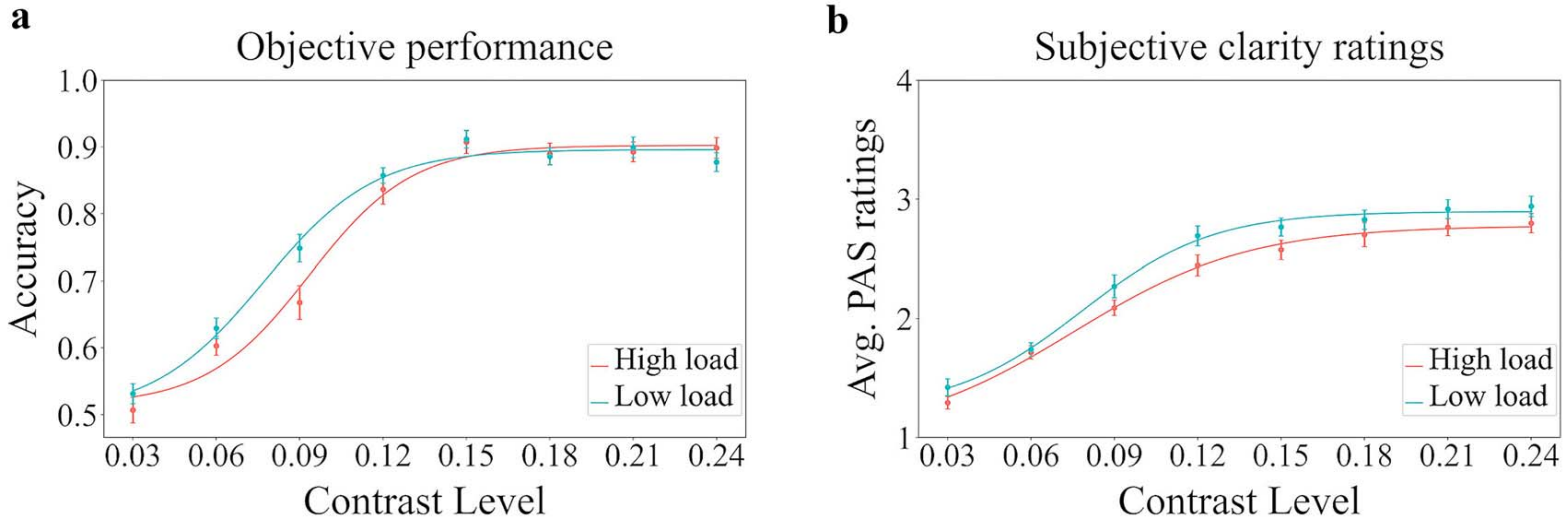
The average accuracy (objective task) and average subjective PAS ratings (subjective task) (grouped data) in high load and low load conditions fitted with a four-parameter nonlinear function. (a) Average accuracy at each contrast level of the scene-images. (b) Average PAS ratings at each contrast level of the scene-images. The error bars are for 95% CI.

**Figure 3:**
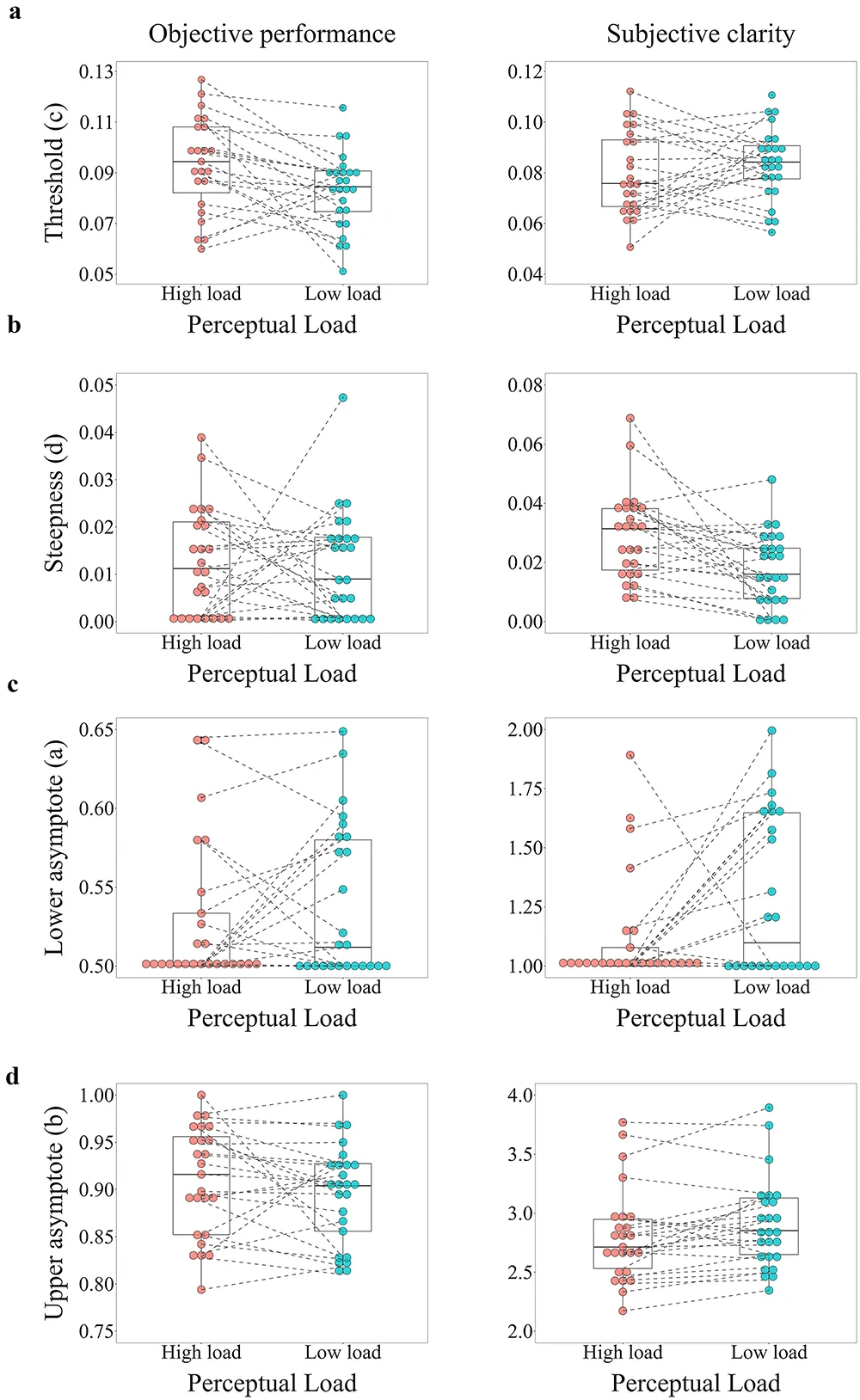
Subject-wise distribution of (a) threshold (*c*), (b) steepness parameter (*d*), (c) lower asymptote (*a*), and (d) upper asymptote (*b*) parameters for objective performance and mean PAS ratings in the high load and low load task conditions.

**Table 1:** Summary statistics (mean and 95%CI) for objective performance and PAS ratings for Experiment 1.

Response	Parameters		Load		
		High			Low
		Mean	CI (95%)	Mean	CI (95%)
Objective performance	*a*	0.528	[0.510, 0.546]	0.539	[0.520, 0.558]
	*b*	0.909	[0.886, 0.932]	0.895	[0.875, 0.916]
	*c*	0.097	[0.088, 0.107]	0.083	[0.077, 0.089]
	*d*	0.012	[0.008, 0.017]	0.012	[0.007, 0.016]
Subjective clarity ratings	*a*	1.117	[1.022, 1.211]	1.330	[1.180, 1.481]
	*b*	2.804	[2.648, 2.960]	2.905	[2.754, 3.055]
	*c*	0.082	[0.074, 0.089]	0.084	[0.078, 0.089]
	*d*	0.029	[0.023, 0.035]	0.018	[0.013, 0.022]

Paired sample *t*-tests were conducted to check the effects of load on the *a, b, c*, and *d* parameters of the curve. Bonferroni correction was used to correct for multiple comparisons, and the corrected *P*-values are reported below. The threshold contrast for identifying scenes for the low load condition was significantly lower than that for the high load condition *t*(24) *=* 3.41, *P* = .009, *d =* 0.68. The parameter *d* was not significantly different between low-load and high-load conditions with, *t*(24) *=* 0.21, *P* = 1, *d =* 0.04. Furthermore, the lower asymptote, *t*(24) *= −*1.29, *P* = 0.82, *d =* 0.25 as well as upper asymptote parameters, *t*(24) *=* 1.26, *P* = .89, *d =* 0.25 were also not significantly different between low load and high load conditions.

### Subjective measure results

#### Overall clarity ratings

The overall mean PAS rating for the low load condition (*M =* 2.43*, SD =* 0.33) was significantly higher than high load condition (*M =* 2.29*, SD =* 0.31), (*t*(24) = −4.78, *P* < .001, *d* = 0.96).

#### Curve fitting results for subjective clarity ratings

The average subjective PAS clarity ratings provided by the participants at various contrast levels for high load and low load conditions were fitted with the four-parameter model (equation 1). The average goodness of fit values for the fitted curves were satisfactory for both high load (group mean *R^2^* = 0.93, *SD =* 0.05) as well as low load condition (group mean *R^2^* = 0.94, *SD =* 0.04). Figure 2B displays the psychometric curves for the average PAS ratings (for all participants) as a function of Contrast for high and low load conditions. The results for the average threshold *c*, steepness of slope *d,* lower asymptote *a*, and upper asymptote *b* parameters for the subjective clarity ratings in the high load and low load conditions are also shown in Fig. 3a–d.

For subjective ratings, paired sample *t*-tests were conducted to check the effects of load on the *a, b, c*, and *d* parameters of the curve (see Table 1 for mean and SD values of the parameters). Bonferroni correction was used to correct for multiple comparisons, and the corrected *P*-values are reported below. The *d* parameter for the high-load condition was significantly higher compared to that of the low-load condition *t(*24*) =* 3.295, *P =* .012, *d =* 0.66. Threshold between high and load conditions was not significantly different, *t(*24*) = −*0.54, *P* > .99, *d* = 0.11. The lower asymptote for high load condition was significantly different from that for low load condition, *t(*24*) =* −2.76, *P =* .043, *d* = 0.55. The upper asymptote for high load was not significantly different from that for the low load condition, *t(*24*) =* −2.29, *P =* .12, *d* = 0.46).

The smaller *d* value for subjective clarity ratings in the low load condition shows that subjective perception of the stimuli during low load condition was more nonlinear and less graded in nature. The gradedness was more in the high load condition. There were no threshold differences.

The correlations between the parameters of the objective performance fits and subjective ratings fits of low and high load conditions were not significant (Bonferroni corrected): (i) low load: (a) threshold: *ρ* = .04, *P* = .84, (b) steepness parameter *d*: *ρ* = −.05, *P* = .82, (c) lower asymptote: *ρ* = −.25, *P* = .23, and (d) upper asymptote: *ρ* = .27, *P* = .18, and (ii) high load, (a) threshold: *ρ* = .06, *P* = .76, (b) steepness parameter *d*: *ρ* = .17, *P* = .42, (c) lower asymptote: *ρ* = −.23, *P* = .26, and (d) upper asymptote: *ρ* = .24, *P* = .25. The results indicate that the parameters for the subjective PAS ratings fits are probably not influenced by corresponding parameters for the objective accuracy fits.

We also performed a nonlinear mixed-effects modelling analysis with curve-type as a within subjects fixed factor and random effects corresponding to subject and curve (accuracy and awareness) within subjects included for each of the parameters, *a, b, c*, and *d* based on the analysis done by Sandberg et al. (2011). The analysis was performed for three parameters for each load type separately (total six parameters) in Experiment 1 since the analysis did not converge for the four-parameter model with the given data (results are shown in Tables S5 and S6 as well as in Figure S3 of the supplementary material). The analysis was performed for each load type separately since the model with load also as a random factor did not converge. Unlike Sandberg et al. (2011), the difference between the threshold parameter for accuracy and awareness was not significantly different. The result indicates that there is no statistical evidence for unconscious processing in the low load condition. The difference between the threshold parameter for accuracy and awareness was significantly different in the high load condition. However, the threshold difference is negative, indicating that awareness does not lag accuracy even in the high load condition. In fact, awareness led accuracy. Awareness is more graded than accuracy as in Sandberg et al. (2011) in both the low load and the high load conditions.

The nonlinear mixed modelling analysis was also performed with load-type as a within subjects fixed factor and subjects as a random factor for all the four parameters of each curve-type separately (total eight parameters). The results (Tables S1 and S2 as well as Figure S1 in the supplementary material) showed a significant effect of load on threshold with accuracy and a significant effect on the two asymptotes and slope with PAS ratings. Except for the significant result with upper asymptote of PAS ratings, rest of the results were similar to that obtained with *t*-tests performed with parameters from curve fits from individual participants.

#### ROC AUC analysis

We did the AUC analysis (based on the method used by Schwiedrzik et al. (2011)), which is a useful way to know if PAS ratings are used meaningfully in an experiment. The results in Experiment 1 showed that mean AUC values were significantly above chance (0.5) in both the low-load condition (*M* = 0.685, *t*(24) = 13.19, *P* < .001) and the high-load condition (*M* = 0.679, *t*(24) = 14.34, *P* < .001), indicating that PAS ratings reliably tracked objective performance. A paired-samples *t-*test revealed no significant difference in AUC between load conditions (*t*(24) = 0.44, *P* = .661), suggesting that the load manipulation did not alter the relationship between subjective visibility and objective accuracy.

#### Perceptual awareness scale rating frequency analysis

To further understand the observed differences in graded nature of awareness, we analyzed the PAS rating value distribution for high load and low load conditions. Figure 4 shows the distribution of PAS rating distributions for each value as a function of load. A repeated measures ANOVA was conducted for each PAS rating value with contrast and load as factors.

**Figure 4:**
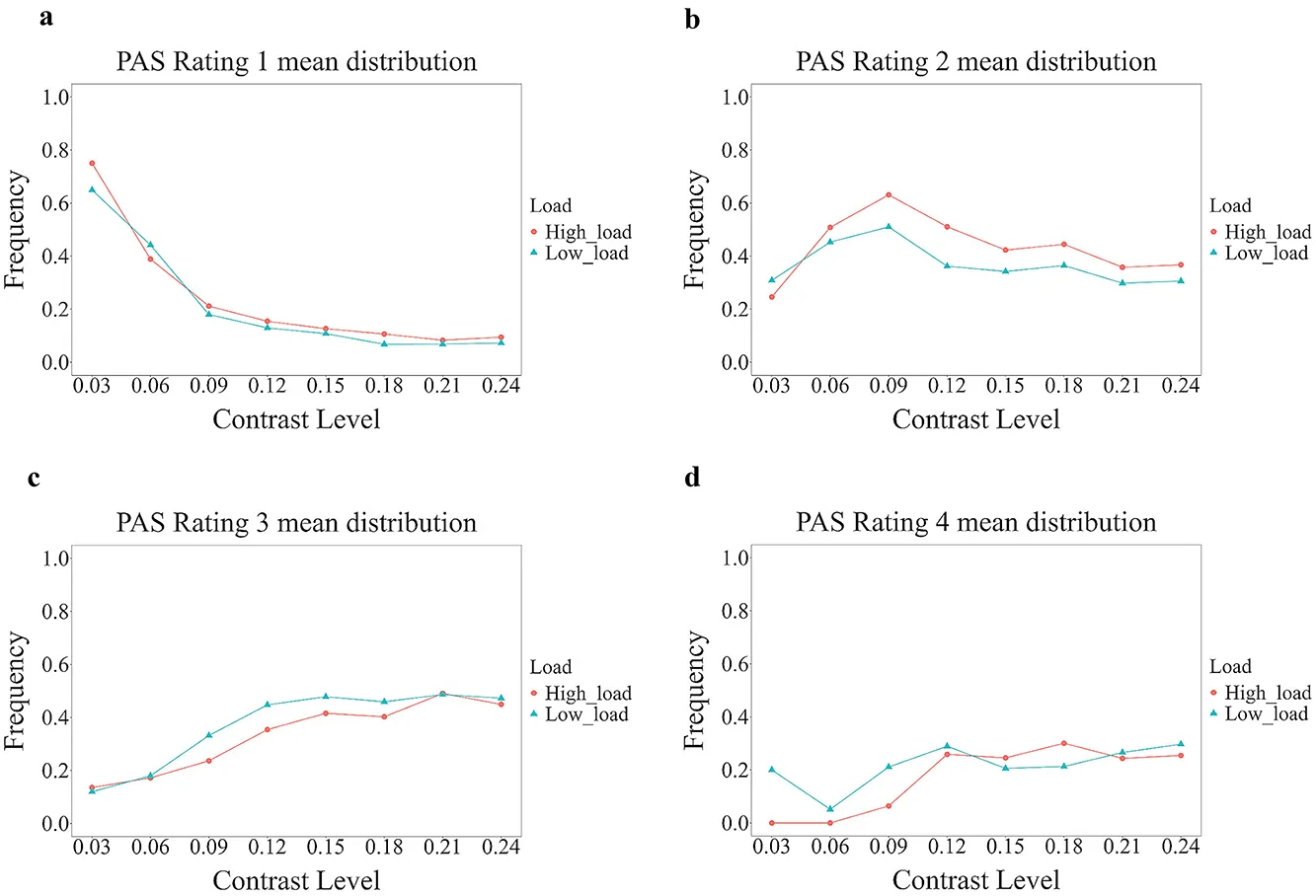
Distribution for each PAS rating value as a function of load and contrast level in Experiment 1: (a) distribution for PAS rating 1, (b) distribution for PAS rating 2, (c) distribution for PAS rating 3, and (d) distribution for PAS rating 4.

*PAS rating 1 analysis:* The ANOVA revealed a significant main effect of contrast, *F*(7, 168) = 172.52, *P* < .001, *η_p_*^2^ = .878, with proportion of PAS 1 values decreasing with increase in contrast (Fig. 4a). The proportion of PAS 1 values for 0.09 contrast was higher than those for higher contrasts (*P*s < .05). In addition, PAS 1 proportion for 0.06 contrast was higher than that for 0.09 and the largest value was for the lowest contrast of 0.03, which was significantly higher than that for 0.06 (*P*s < .001). The main effect of load was not significant, *F*(1, 24) = 1.79, *P* = .194, *η_p_*^2^ = .069. The interaction between contrast and load was also not significant, *F*(7, 168) = 1.62, *P* = .133, *η_p_*^2^ = .063.

*PAS rating 2 analysis:* The ANOVA revealed significant a main effect contrast, *F*(7, 168) = 11.05, *P* < .001, *η_p_*^2^ = .315 (Fig. 4b). Bonferroni-corrected pairwise comparisons for the main effect of contrast showed that PAS 2 responses were significantly less frequent at contrast level 0.03 than at contrast levels 0.06 and 0.09 (both adjusted *Ps* < .001). In turn, PAS 2 responses were significantly more frequent at contrast level 0.09 than at contrast levels 0.12, 0.15, 0.18, 0.21, and 0.24 (all adjusted *Ps* ≤ .05). No other pairwise comparisons reached significance after correction.

There was significant effect of load, *F*(1, 24) = 21.39, *P* < .001, *η_p_*^2^ = .471 with a higher proportion of PAS 2 responses in the high load condition compared to the load condition. The interaction between contrast and load was significant, *F*(7, 168) = 2.96, *P* = .006, *η_p_*^2^ = .110, indicating that the effect of load on PAS 2 response proportions differed as a function of contrast. Significant differences between high load and low load conditions were observed at middle and higher contrast levels (0.09, 0.12, 0.15, 0.18, and 0.21; all *P*s < .05), with higher proportion of PAS rating 2 responses under high load. A similar trend was also seen at 0.24 contrast but was not significant (*P* = .085). These were part of middle contrast levels and is the possible reason for a slope difference between the two load conditions. No significant differences between load conditions were observed at low contrast levels of 0.03 and 0.06 (*Ps* > .05).

*PAS rating 3 analysis:* The ANOVA revealed significant main effects of contrast, *F*(7, 168) = 38.66, *P* < .001, *η_p_*^2^ = .617. PAS 3 responses increased as contrast increases for low contrasts and asymptote at contrast of 0.12 (Fig. 4c). Bonferroni-corrected pairwise comparisons showed that PAS 3 proportions were more for higher contrasts (greater than equal to 0.12) compared to 0.03, 0.06, and 0.09 (*P*s < .05). The main effect of load was significant, *F*(1, 24) = 6.32, *P* = .019, *η_p_*^2^ = .208 with lesser PAS 3 responses in the high load condition. The interaction between contrast and load was not significant, *F*(7, 168) = 0.94, *P* = .476, *η_p_*^2^ = .038, indicating that the effect of contrast on PAS 3 responses was comparable across load conditions.

*PAS rating 4 analysis:* The ANOVA for the proportion of PAS rating 4 responses showed a significant main effect of contrast, *F*(7, 168) = 16.33, *P* < .001, *η_p_*^2^ = .405, indicating that the proportion of PAS 4 responses varied across contrast levels (Fig. 4d). Bonferroni-corrected pairwise comparisons for Contrast showed that PAS 4 proportions were significantly lower at contrast levels 0.03, 0.06, and 0.09 compared to the remaining higher contrast levels (adjusted *Ps* ≤ .05). There was also a significant main effect of load, *F*(1, 24) = 13.10, *P* = .001, *η_p_*^2^ = .353 with higher proportion of PAS 4 responses in the low load condition. The interaction between contrast and load was not significant, *F*(7, 168) = 0.91, *P* = .499, *η_p_*^2^ = .037.

Overall, the PAS distribution analyses for Experiment 1 indicate that the proportion of PAS 1 rating responses decreased as contrast increased as one would expect. There were not that many PAS 4 responses even at the highest contrast we used and there were mostly PAS 3 responses at the higher contrast levels. PAS 3 responses were low at low contrasts and increased with contrast but did not show a significant load effect or interaction with load effect. PAS 2 rating responses increased and then decreased a bit with increase in contrast, and they showed an interaction between load and contrast with more PAS 2 responses in the high load condition compared to the load condition indicating the intermediate visibility level of brief glimpse is what seems to be influenced by the load manipulation.

## Discussion

These results show that gist perception is graded with gradedness dependent on perceptual load. Awareness of gist was more graded in the high load condition compared to the low load condition providing support for theories of consciousness that postulate gradedness of visual awareness (Windey and Cleeremans 2015, Fazekas and Overgaard 2016, 2018, Lamme 2010).

The current results extend findings of Lavie et al. (2014) by showing that contrast response function for scene perception during high load shifts rightwards (Fig. 2a), like that obtained earlier with Gabor patches as a function of load. However, it is important to mention that unlike only objective performance data (Lavie et al. 2014), we had both objective performance as well as subjective ratings data in the current experiment. In terms of proposals linking load to awareness, the current findings show that awareness of scene gist is probably not discrete. Scene perception is graded and gradedness is a function of perceptual load. Changes in load and hence attentional resources for the scene gist perception task possibly influence the clarity or precision of perceptual representations of scenes not just perceptual sensitivity.

Furthermore, the current results provide clarity to the ongoing debate regarding whether gist perception is independent of attention (Li et al. 2002, Koch and Tsuchiya 2007, Cohen et al. 2011, Mack and Clarke 2012). Our current results do indicate that attention as conceived as attentional resource associated with task demands does influence gist perception. Performance in gist perception task was better when participants perform a concurrent low load task compared to a high load task. More importantly, the novel result here is that the dependence of gist perception on available attentional resources is graded as indicated by slope changes in function fits for PAS ratings.

Given that the perceptual load in the concurrent task influences the gradedness of scene perception in the secondary task, it is important to elucidate the mechanism that underlies this change in gradedness of gist perception as a function of load. One potential way to address this issue is by using the external noise paradigm developed to elucidate mechanisms underlying exogenous and endogenous attention (Lu and Dosher 1998). Experiment 2 addresses this issue.

## Experiment 2

Building on the noisy Perceptual Template Model (PTM) of a human observer, Lu and Dosher (1998, 2005) proposed that attentional mechanisms like stimulus enhancement and external noise suppression can be identified by manipulating external noise added to a stimulus. They argued that these mechanisms efficiently operate only at specific noise levels and increase perceptual sensitivity, with stimulus enhancement being more effective at low noise levels and external noise suppression at high noise levels. Hence, they argued that if attention operates through amplification, its effect on sensitivity should appear only at low noise levels. If attention operates through noise suppression, its effect should appear at high noise levels. We reasoned that this argument is applicable not just for thresholds but even when the perceptual performance measure is accuracy. If a system (e.g. like a radio) receives a signal with high levels of noise, then amplification would not help since the noise will also get amplified, making signal detection harder. In this case, a filter or noise suppression would be more effective. If the noise levels are low, then increasing the volume would help since the amplification effect on signal would be more than that for the noise at low levels of noise. This argument is not dependent on the system operating at threshold levels and should apply to performance, in general. Hence, we decided to manipulate noise levels to identify the potential mechanism through which perceptual load influence gist perception performance in Experiment 2.

Experiment 2 applied this logic to the load manipulation used in Experiment 1. Instead of manipulating scene contrast, we added varying levels of external Gaussian noise to the scenes, allowing us to ask whether the effect of load on scene perception reflects amplification or noise suppression. Extending the argument made by Lu and Dosher (1998, 2005), a load-related change confined to the low noise levels or the upper asymptote of the accuracy function would indicate stimulus enhancement. A load-related change confined to high noise levels or the lower asymptote of the accuracy function would indicate noise suppression. To test whether the same mechanism also drives load-related changes in subjective awareness, we examined the correlation between load-related changes in the relevant asymptote of the accuracy function and load-related changes in the slope of the PAS function. Given prior links between perceptual load and suppression mechanisms (Gutteling et al. 2022, Lavie et al. 2014, Parks et al. 2013), we expected this correlation to emerge primarily for the lower asymptote.

## Method

### Participants

Forty-two naïve volunteers (*M*_age_ = 22.42 years, *SD* = 3.2 years, females = 15) with normal or corrected-to-normal vision participated in the current study after providing informed consent. We used GPower 3.1 and computed required sample size for a desired correlation of 0.4 between differences in lower asymptote of accuracy fits and differences in slope of PAS ratings fits, *α* = 0.05, and power = 0.8. The required sample size was 37. Data from five participants were removed because of bad fit either in the objective performance or the subjective-rating curve fits (*R*^2^ < 0.80) resulting in data from 37 participants. They were all provided with monetary compensation of 100/−. The study was approved by the Institutional Ethics Committee.

### Stimuli and apparatus

The stimuli were similar to Experiment 1 except that instead of images with different contrasts, images with different amounts of external Gaussian noise were used. The standard deviation of the different levels of the added external noise in the images were 10, 30, 50, 70, 90, 110, 130, 150, and 170. The apparatus was the same as in Experiment 1.

### Procedure

The procedure of this experiment was similar to Experiment 1 (Fig. 5). Participants performed both load and gist perception and reported their subjective clarity rating similar to Experiment 1. Overall, the experiment consisted of 360 trials (two blocks of high load condition and two blocks low load condition with 90 trials each). The rest of the details were the same.

**Figure 5:**
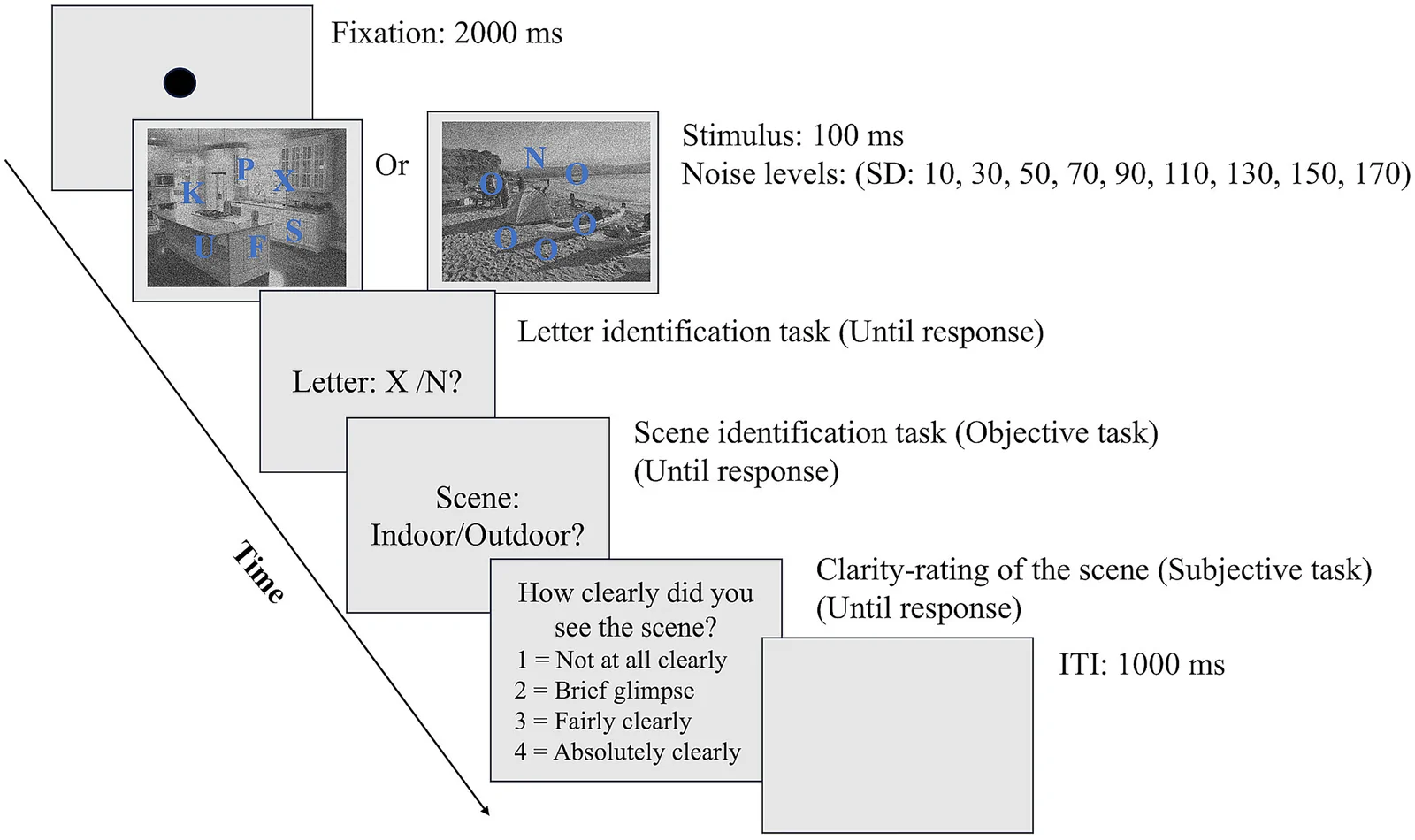
Trial structure of Experiment 2.

### Data analysis

The data analysis protocol was the same as that in Experiment 1.

## Results

### Letter identification accuracy

As expected, accuracy in the letter identification task was significantly less in the high load condition (*M* = 75.48%, *SD* = 9.12) compared to the low load condition (*M* = 87.92%, *SD* = 7.19) condition, *t*(36) = 11.21, *P* < .001, *d =* 1.84 indicating that the load manipulation was effective.

### Scene perception results

#### Overall scene identification accuracy

Overall scene identification accuracy was more in the low load (*M* = 79.67%, *SD* = 7.12) compared to the high load (*M* = 76.57%, *SD* = 7.61) condition, *t*(36) = 6.26, *P* < .001, *d =* 1.03 indicating that the high load condition restricted the identification of scene gist information more compared to the low load condition. The overall proportion of correctly identified indoor/outdoor scenes were the same: 78.13% in indoor category and 78.10% in outdoor category. A paired *t-*test showed no difference in accuracy for indoor and outdoor scenes, *t*(36) = 0.02, *P* = .98, *d =* 0.001.

#### Curve fitting results for scene identification accuracy

The four-parameter psychometric model (equation 1) was fitted to the individual subject’s objective performance, i.e. mean accuracy in identifying indoor/outdoor scenes for high and low perceptual load conditions at different levels of external noise. Curve fits for group mean data are shown in Fig. 6. The nonlinear model provided a good fit for the individual subjects’ performance data with group mean *R*^2^ = 0.90 (*SD =* 0.05*)* for the low load condition and group mean *R*^2^ = 0.91 (*SD =* 0*.*04*)* for the high load condition. The results for threshold, slope*,* and lower asymptote parameters for the objective task performance and subjective ratings for high and low load conditions are presented in Fig. 7a–c. Summary statistics for all the parameters are given in Table 2.

**Figure 6:**
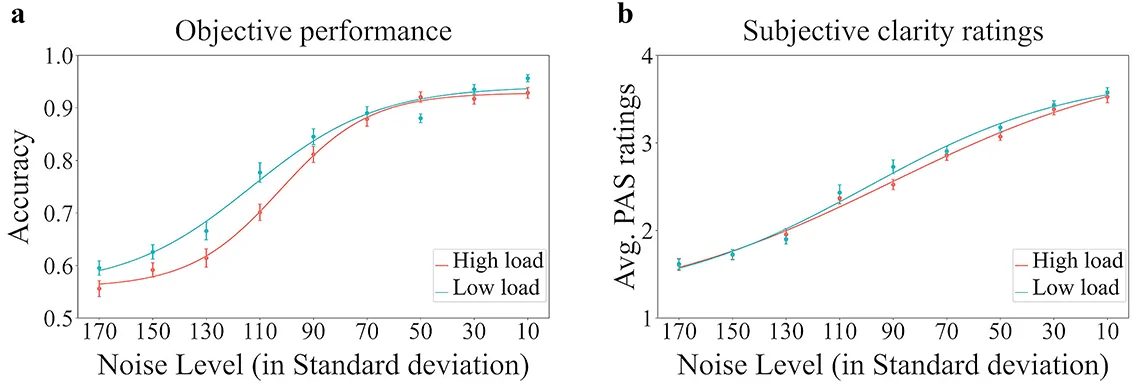
The average accuracy (objective task) and average subjective PAS ratings (subjective task) (grouped data) in high load and low load conditions fitted with a four-parameter nonlinear function. (a) Average accuracy at each noise level of the scene-images. (b) Average PAS ratings at each noise level of the scene-images. Noise levels are plotted in decreasing order on x-axis in both a and b. The error bars provided are 95% CI.

**Figure 7:**
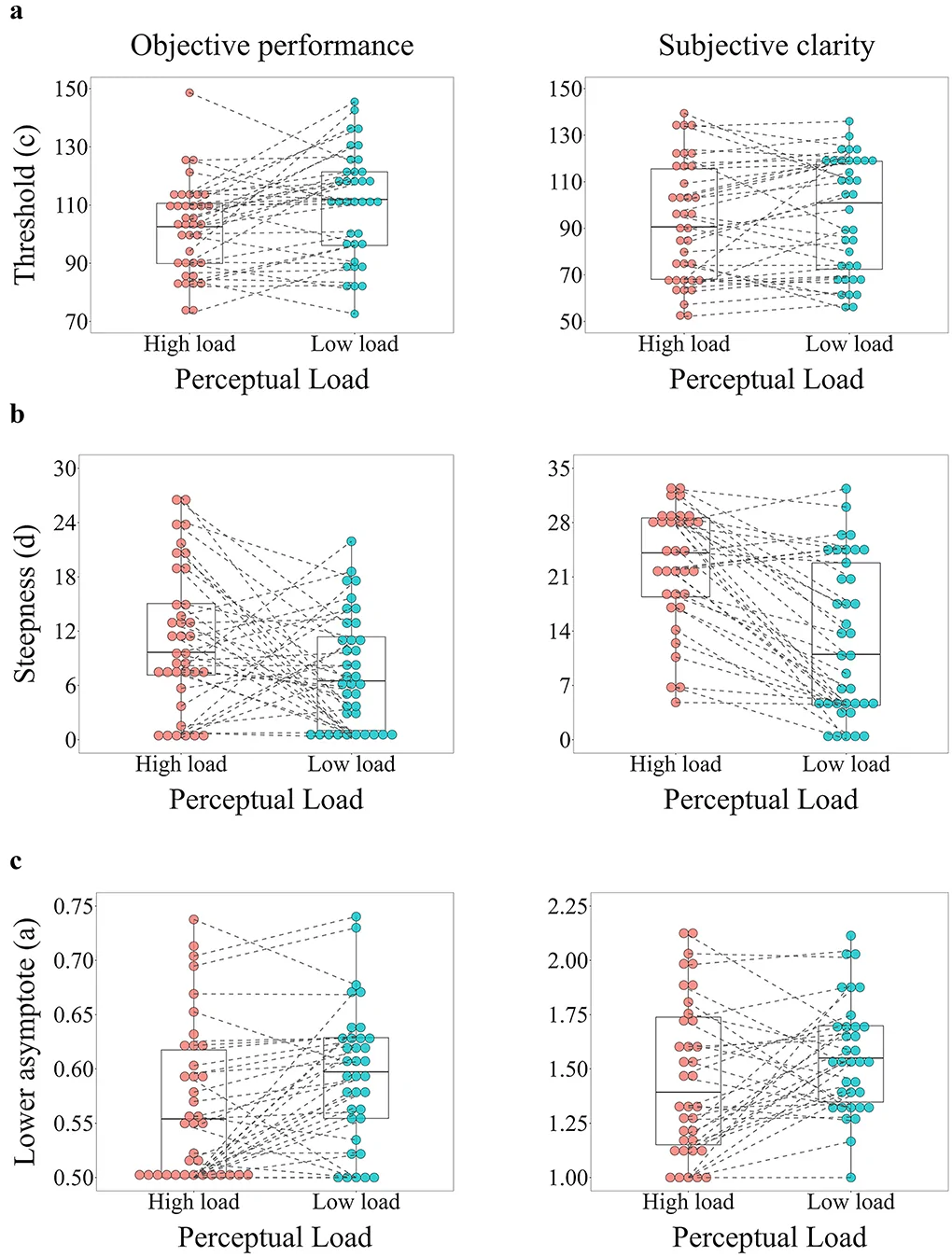
Subject-wise distribution of (a) threshold, (b) slope, and (c) lower asymptote parameters of the fitted functions of objective performance and mean PAS subjective ratings for the high load and low load task conditions.

**Table 2:** Summary statistics (mean and 95% CI) for objective performance and subjective clarity ratings for Experiment 2.

Response	Parameters	Load			
		High		Low	
		Mean	CI (95%)	Mean	CI (95%)
Objective performance	*a*	0.921	[0.909, 0.933]	0.918	[0.907, 0.928]
	*b*	0.568	[0.545, 0.591]	0.603	[0.580, 0.627]
	*c*	102.085	[97.000, 107.169]	109.20	[102.925, 115.475]
	*d*	11.175	[8.661, 13.690]	7.48	[5.489, 9.472]
Subjective clarity ratings	*a*	3.582	[3.439, 3.725]	3.505	[3.381, 3.629]
	*b*	1.503	[1.365, 1.641]	1.636	[1.516, 1.756]
	*c*	92.215	[83.929, 100.500]	94.223	[85.802, 102.645]
	*d*	24.342	[21.345, 27.339]	13.005	[9.797, 16.213]

Paired sample t-tests were conducted to check the effects of high and low load conditions on the *a, b, c*, and *d* parameters of the curve. Bonferroni correction was used for *P*-value correction for the four tests and corrected *P*-values are reported below. The *t*-tests showed that noise threshold for identifying scenes during the low-load condition was significantly higher than the high-load condition, *t*(36) *=* 2.82, *P* = .03, *d =* 0.46. This shows that participants can correctly identify scenes during low load condition despite large external noise, i.e. performance under low load can accommodate high amounts of external noise. There was no significant difference for steepness parameter *d* between low-load and high-load conditions, *t*(36) *=* 2.19, *P* = .14, *d =* 0.36. Furthermore, the *b* parameter (lower asymptote in this experiment) for the high load condition was significantly lower compared to the low load condition, *t*(36) = 3.67, *P* = .003, *d* = 0.604. This shows that performance decreased for the high load condition compared to the low load condition when external noise is higher (Fig. 6a). However, the difference between the high load and low load conditions for the *a* parameter was not significant, *t*(36) = 0.58, *P* = 1, *d* = 0.09, which indicates lack of difference when noise levels are low.

The results show that the mechanism underlying the effect of load in this study is due to external noise suppression (Lu and Dosher 2005). Lu and Dosher (2005) argued that if the nature of the attentional mechanism is to decrease external noise, then that should result in effects of attention being visible with high levels of external noise, i.e. a change in the lower asymptote parameter (if noise is plotted with decreasing levels of noise).

### Subjective measure results

#### Overall clarity ratings

The overall mean PAS rating for the low load condition (*M* = 2.59, *SD* = 0.22) was not significantly different than high load condition (*M* = 2.56, *SD* = 0.28), (*t*(36) = −1.17, *P* = .25, *d* = 0.19).

#### Curve fitting results for subjective clarity ratings

The average subjective PAS clarity ratings provided by the participants at various noise levels for high load and low load conditions were fitted with the four-parameter model (equation 1). The average goodness of fit values for the fitted curves were good for both high load (group mean *R^2^* = 0.95, *SD =* 0.04) as well as low load condition (group mean *R^2^* = 0.96, *SD =* 0.03). Figure 6b displays the fitted curves for the average PAS ratings (for all participants) as a function of noise levels for high and low load conditions. The results for the average threshold, slope*,* and lower asymptote for the subjective clarity ratings in the high load and low load conditions are shown in Fig. 7b.

For subjective ratings fit as well, we conducted paired sample t-tests to check the effects of load on the *a, b, c*, and *d* parameters (see Table 2 for summary statistics). Bonferroni correction was used for multiple comparisons and corrected *P*-values are reported below. The *d* parameter for the high load condition was significantly higher compared to the low load condition, *t*(36) = 6.35, *P* < .001, *d* = 1.04. The result indicates that the slope is lower in the high load condition and visual awareness is more graded. The threshold (*c* parameter) for the high load condition was not significantly different compared to the low load condition, *t*(36) = 0.74, *P* = 1, *d* = 0.12. Furthermore, the lower asymptote for the high load condition was not significantly different compared to the low load condition, *t*(36) = 2.20, *P* = .13, *d* = 0.36. There was also no significant difference for the upper asymptote between the high load and low load conditions, *t*(36) = 1.79, *P* = .33, *d* = 0.29.

The results show smaller *d* value for low load condition indicating that subjective perception of the stimuli during the low load condition was more nonlinear and discrete in nature. On the other hand, there was a graded experience of the stimuli during the high load condition reflected by higher values of *d* value for the high load condition.

The correlations between the parameters of the objective performance fits and subjective ratings fits of corresponding load conditions were not significant (Bonferroni corrected, *P* > .05 for all parameters): (i) low load, (a) threshold: *ρ* = .14, *P* = .42, (b) steepness parameter *d*: *ρ* = .01, *P* = .95, (c) lower asymptote: *ρ* = .02, *P* = .92, (d) upper asymptote: *ρ* = .12, *P* = .48, and (ii) high load, (a) threshold: *ρ* = .05, *P* = .77, (b) steepness parameter *d*: *ρ* = −.13, *P* = .43, (c) lower asymptote: *ρ* = .28, *P* = .09, (d) upper asymptote: *ρ* = .41, *P* = .01 (Bonferroni corrected *P* = .098). Similar to Experiment 1, the lack of correlations between the parameters from objective and subjective fits indicate they may not significantly influence each other.

We also performed a nonlinear mixed-effects modelling analysis with curve-type as a within subjects fixed factor and random effects corresponding to subject and curve (accuracy and awareness) within subjects included for each of the parameters, *a, b, c*, and *d* based on the analysis done by Sandberg et al. (2011). The results are presented in Tables S7 to S8 as well as Figure S4 of the supplementary material. The analysis was performed for each load type separately since the model with load also as a random factor did not converge. Unlike Sandberg et al. (2011) but similar to Experiment 1, the difference between the threshold parameter for accuracy and awareness was significantly different but with awareness leading accuracy. Similar effect was obtained with threshold parameter in the high load condition. The results do not provide any evidence for unconscious processing in both the load conditions when external noise was manipulated in Experiment 2 (similar to Experiment 1). Awareness is more graded than accuracy as in Sandberg et al. (2011) in both the low load and the high load conditions again similar to Experiment 1.

The nonlinear mixed modelling analysis was also performed with load-type as a within subjects fixed factor and subjects as a random factor for all the four parameters of each curve-type separately (total eight parameters). The results (Tables S3 and S4 as well as Figure S2 in the supplementary material) once again showed a significant effect of load on threshold and lower asymptote, with accuracy. The results also showed a significant effect on both asymptotes and slope with PAS ratings. The threshold and asymptote effect with accuracy as well as the slope effect with PAS ratings are consistent with the earlier analysis with t-tests performed with parameters from curve fits from individual participants.

#### ROC AUC analysis

For Experiment 2 as well, we found mean AUC values were significantly above chance level (0.5) in both the low-load condition (*M* = 0.68, *SD* = 0.05), *t*(36) = 20.44, *P* < .001, and the high-load condition (*M* = 0.69, *SD* = 0.06), *t*(36) = 20.28, *P* < .001. These findings indicate that PAS ratings reliably tracked objective performance in both load conditions. A paired-samples *t-*test revealed no significant difference in AUC between the low-load and high-load conditions, *t*(36) = −0.84, *P* = .409, suggesting that perceptual load did not significantly alter the relationship between subjective visibility ratings and objective scene-discrimination accuracy. Taken together these findings show that participants used the PAS scale in a meaningful manner and perceptual load did not alter the relationship between subjective visibility and objective performance.

#### Perceptual awareness scale rating frequency analysis

To further understand the observed differences in graded nature of awareness, we analyzed the PAS rating value distribution for high load and low load conditions. Figure 8 shows the distribution of PAS rating distributions for each value as a function of load and noise levels. A repeated measures ANOVA was conducted for each PAS rating value with noise levels contrast and load as factors.

**Figure 8:**
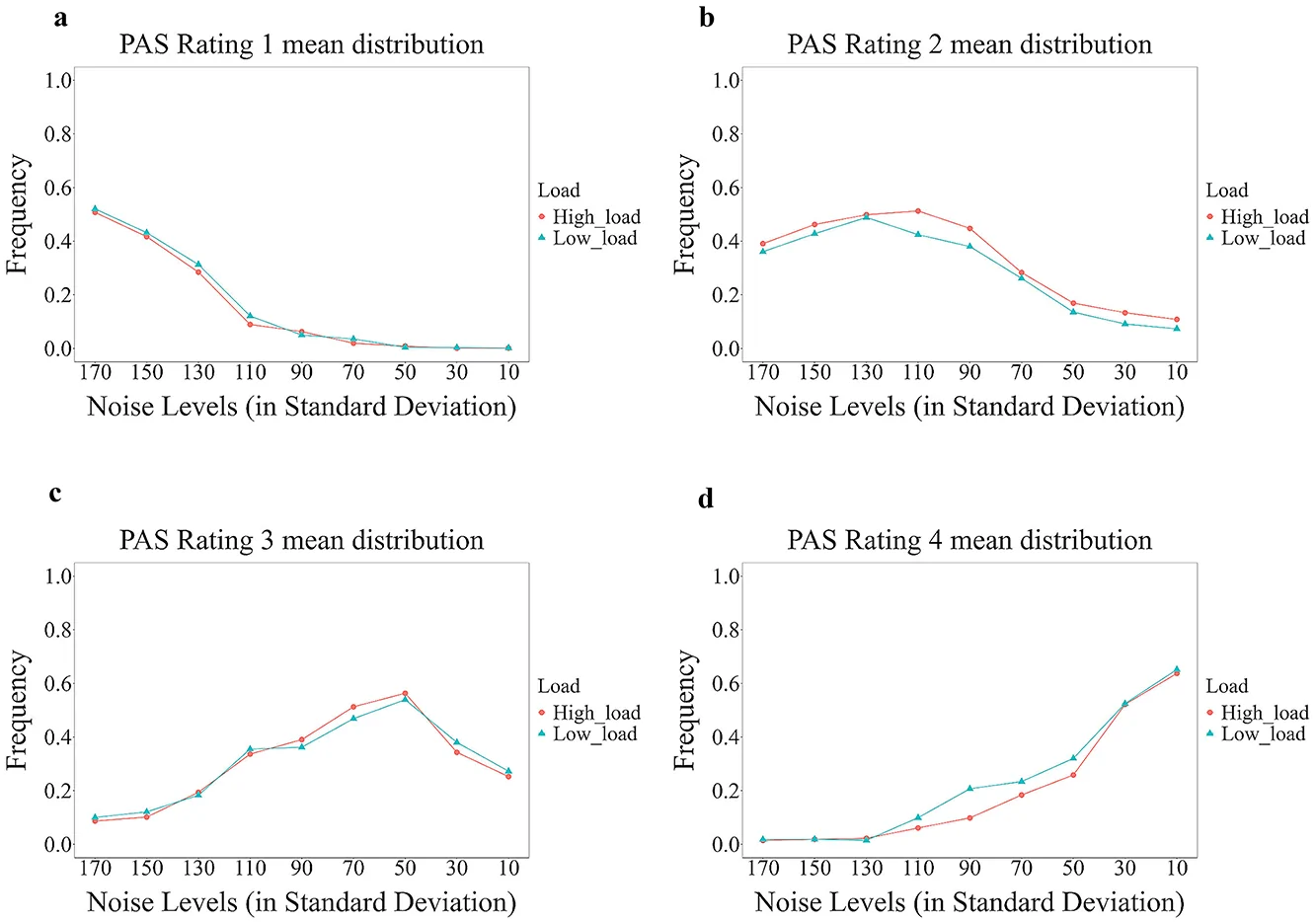
Distribution for each PAS rating value as a function of load and noise level in Experiment 2: (a) distribution for PAS rating 1, (b) distribution for PAS rating 2, (c) distribution for PAS rating 3, and (d) distribution for PAS rating 4.

*PAS rating 1 analysis:* There was a significant main effect of noise, *F*(8, 288) = 144.23, *P* < .001, *η_p_*^2^ = .800, with proportion of PAS 1 ratings decreasing with decrease in noise levels (Fig. 8a). PAS 1 proportions were more for noise levels 170–110 compared to all the noise levels below 110 (*P*s < .05). For the range of noise levels 170 to 110, the proportion of PAS 1 proportions were higher for the higher contrast value (*P*s < .05). The main effect of load was not significant, *F*(1, 36) = 1.02, *P* = .320, *η_p_*^2^ = .028. The interaction between noise level and load was also not significant, *F*(8, 288) = 0.46, *P* = .883, *η_p_*^2^ = .013.

*PAS rating 2 analysis:* There was a significant main effect of noise, *F*(8, 288) = 62.44, *P* < .001, *η_p_*^2^ = 0.634. Figure 8b shows that PAS 2 proportions are higher at higher noise levels and then they decrease with decrease in noise (less sharply than that PAS 1 responses). Bonferroni-corrected pairwise comparisons showed that relative to the lowest noise levels (10, 30, 50, and 70), PAS 2 proportions were significantly higher at intermediate noise levels, particularly at noise levels 90, 110, and 130 (*Ps* < .05). PAS 2 proportions were not significantly different across noise levels 90, 110, 130, and 150. However, PAS 2 proportion declined at the highest noise level of 170 compared to noise levels of 150 and 130 (*P*s < .05). The results are somewhat similar to Experiment 1 with more PAS 2 rating responses for intermediate levels and those responses being higher for high load compared to low load condition. The main effect of load was significant, *F*(1, 36) = 9.90, *P* = .003, *η_p_*^2^ = 0.216 with higher PAS 2 proportion in the high load condition. The interaction between noise level and load was not significant, *F*(8, 288) = 0.62, *P* = .758, *η_p_*^2^ = 0.017.

*PAS rating 3 analysis:* There was a significant main effect of noise, *F*(8, 288) = 46.80, *P* < .001, *η_p_*^2^ = 0.565, indicating that PAS 3 responses varied substantially across noise levels (Fig. 8c). The proportion of PAS 3 responses increased with decrease in noise and peaked at the noise level of 50 and then decreased. PAS 3 responses peaked later than PAS 2 responses. Bonferroni-corrected pairwise comparisons showed that relative to the lowest noise levels, PAS 3 proportions increased substantially between noise levels 10 and 50, 10 and 70, and 30 and 50 (*Ps* < .05). PAS 3 responses were also significantly more frequent at noise levels 50 and 70 than at higher noise levels including 130, 150, and 170 (*Ps* < .05). PAS 3 proportions declined at the highest noise levels. Noise levels 130, 150, and 170 yielded significantly lower PAS 3 proportions than noise levels 50, 70, 90, and 110 (*Ps* < .05) Furthermore, PAS 3 responses were significantly less at noise level 150 compared to noise level 130, noise level 170 compared to 130 (*Ps <* .05). No significant difference was observed between noise levels 150 and 170. Neither the main effect of load, *F*(1, 36) < 0.001, *P* = .992, *η_p_*^2^ < 0.001, nor the interaction between noise and load, *F*(8, 288) = 0.84, *P* = .570, *η_p_*^2^ = 0.023, was significant.

*PAS rating 4 analysis:* There was a significant main effect of noise level, *F*(8, 288) = 116.40, *P* < .001, *η_p_*^2^ = 0.764, indicating that PAS 4 proportions varied across noise levels. Overall, proportion of PAS 4 responses increased with decrease in levels of noise (Fig. 8d). Proportion of PAS 4 responses for all the noise levels in the 10–90 range were significantly higher than those between 110 and 170 range (*P*s < .05). Proportion of PAS 4 responses were greater for noise level of 10 compared to 30, 30 compared to 50, 50 compared to 90, and 70 compared to 110 (*P*s < .05). There was also a significant main effect of load, *F*(1, 36) = 4.39, *P* = .043, *η_p_*^2^ = 0.109, with participants reporting more PAS 4 rating responses in the low-load condition than in the high-load condition. The interaction between noise and load was significant, *F*(8, 288) = 2.63, *P* = .008, *η_p_*^2^ = 0.068, indicating that the effect of load on PAS 4 reports was dependent on noise level. Pairwise Bonferroni corrected comparisons examining the effect of load at each noise level indicated that proportions of PAS rating 4 significantly lower in the high load condition than in the low-load condition only at noise levels of 50 and 90 (*P*s < .05).

Overall, the PAS distribution analyses for Experiment 2 indicate that the proportion of PAS 1 rating responses decreased as noise level decreased as one would expect. An opposite pattern, as expected, can be seen with PAS 4 responses with very few PAS 4 responses at high noise levels and as noise levels decreased proportion of PAS 4 responses increased. Both PAS 2 and PAS 3 responses were initially low, then increased with decrease in noise levels and then decreased a bit. PAS 2 responses peaked earlier (with higher noise levels) than PAS 3 responses. Similar to Experiment 1, PAS 2 responses showed an interaction between low load and high load and with medium and slightly lower noise levels, proportion of PAS 2 responses were higher for high load condition compared to the low load condition. Once again, there seems to be a clear and consistent effect of load on the intermediate visibility level of brief glimpse.

#### Correlation between objective performance asymptote at high external noise and slope and threshold parameters of the subjective ratings data

To investigate the relationship between the mechanism underlying objective performance and subjective perceptual ratings, we conducted a separate correlation analysis. More specifically, we examined whether load-related changes in asymptotes of objective performance fits (difference in lower asymptote between the low load and high load conditions as well as difference in upper asymptote between the low load and high load conditions) was associated with corresponding changes in subjective ratings (differences in steepness parameter *d* of PAS ratings fits between the low load and high conditions). There was a significant correlation (after Bonferroni adjustment) between change in lower asymptote of accuracy and change in steepness parameter *d* of subjective ratings (*ρ* = −.39, *P* = .04). The correlation (after Bonferroni adjustment) between change in upper asymptote of accuracy and change in steepness parameter *d* of subjective ratings was not significant (*ρ* = .02, *P* ≈ 1). Together, these results suggest that participants’ ability to suppress high external noise through attentional resources dependent on perceptual load is associated with changes in gradedness of subjective clarity.

## Discussion

As expected, there was a difference in threshold with higher threshold in the low load condition indicating more attentional resources for the scene perception task in the low load condition. The results from Experiment 2 on gradedness in PAS ratings is similar to that of Experiment 1. Visual awareness for scene gist perception is more graded during the high load compared to the low load condition with higher *d* values in the high load condition. There was difference in lower asymptote for accuracy functions indicating that performance is lesser for high load compared to low load when external noise levels are high. This indicates that load operates by suppressing external noise. More importantly, there was a relationship between the difference in lower asymptote between the low load and high load conditions and the difference in steepness parameter between the low load and high load conditions. The more the lower asymptote was higher in a particular load condition, the *d* parameter and hence, gradedness was lower for that load condition. These results indicate that external noise suppression is a potential mechanism that underlies the dependence of gradedness on perceptual load.

## General discussion

The results show that gradedness, indexed by the steepness of the slope of the function fitted for PAS ratings, changes as a function of load in both experiments. Gradedness was greater under high than low load whether contrast (Experiment 1) or external noise (Experiment 2) was manipulated. These findings support theories that treat consciousness as graded (Anzulewicz et al. 2015, Fazekas and Overgaard 2018, Jimenez et al. 2020, Windey et al. 2013, 2014) and are consistent with proposals that assign an explicit role to attention in shaping gradedness (Fazekas and Overgaard 2018).

The load manipulation was effective, as indicated by higher letter identification accuracy under low than high load (Lavie et al. 2004, 2014). Consequently, gist perception thresholds were lower under low load in both experiments, consistent with prior evidence that load influences visual awareness (Cartwright-Finch and Lavie 2007, Lavie et al. 2014). However, the earlier studies used simpler detection or orientation discrimination tasks, whereas our task involved scene gist. In Lavie et al. (2014), load affected threshold but not slope or asymptote, a pattern interpreted as contrast gain. In our data, load affected the slope of objective accuracy in both experiments, indicating that the just-noticeable difference for scene identification also shifts with concurrent task demand, and it affected the lower asymptote under difficult conditions (low contrast or high noise). This broader signature suggests that load may operate through mechanisms beyond contrast gain, including filtering or noise suppression.

Analysis with curve type as a factor (Sandberg et al. 2011) showed that awareness was more graded than accuracy in both experiments, with awareness changing more slowly as a function of contrast and noise than accuracy, mirroring earlier findings. The relationship between awareness and accuracy, however, was not fixed. In both Experiments 1 and 2, awareness did not lag accuracy and actually led accuracy in three out of four conditions, contrary to Sandberg et al. (2011). Whether awareness lags or leads accuracy therefore appears to depend on stimulus and attentional factors, and future models of gradedness will need to accommodate this variability.

### Mechanism influencing gradedness of gist perception

Several mechanisms have been proposed to underlie attentional effects on visual perception, including filtering (Broadbent 1958), signal amplification (Fazekas and Nanay 2020, Lu and Dosher 1998), and external noise suppression (Lu and Dosher 1998). Using the PTM with external noise manipulation, Lu and Dosher (1998, 2005) attributed exogenous cuing effects to amplification and noise reduction, and endogenous cuing effects to amplification. Other attentional manipulations have been shown to affect resolution through signal enhancement or filtering (Carrasco 2011, Carrasco and Barbot 2019). For perceptual load specifically, contrast gain has been proposed as the mechanism underlying effects on visual awareness (Lavie et al. 2014).

The multi-factor account of awareness (Fazekas and Overgaard 2018) holds that conscious perception depends on the intensity, precision, and temporal stability of representations at each level of processing, with attentional amplification shaping intensity, attentional tuning shaping precision, and recurrent processing shaping stability. Stimuli with fewer allocated resources are represented by low-quality population codes and experienced only faintly. On this view, the high load condition in our study, in which the letter task consumed more resources, should leave scene representations less precise, yielding more graded awareness.

Most prior work on the mechanisms underlying attentional effects on awareness has used feature or object recognition tasks. By using external noise in Experiment 2, we extended this approach to gist perception. The PTM provides a direct rationale for the effects of external noise on objective performance but not on PAS ratings, so we examined the correlation between load-related changes in objective performance and load-related changes in gradedness. The significant correlation suggests that the mechanism driving the effect of load on performance may also drive the change in gradedness, at least for gist perception. Whether the same mechanism, or additional ones, underlies other gradedness effects reported in the literature (Windey et al. 2013, 2014, Anzulewicz et al. 2015, Jimenez et al. 2020, Thiruvasagam and Srinivasan 2021) remains an open question.

### Implications for gradedness theories of consciousness

The current findings challenge accounts that argue visual awareness is always all-or-none (Dehaene et al. 2003, Sergent and Dehaene 2004, Vugt et al. 2018) and support graded accounts such as the Recurrent Processing Hypothesis (Lamme 2010) and the Radical Plasticity Thesis (Cleeremans 2011, Cleeremans et al. 2020). That gradedness in a secondary scene task varied with load in the primary task shows that attentional processes shape gradedness (Fazekas and Overgaard 2018, Overgaard 2017), consistent with prior evidence that spatial scope of attention influences gradedness (Thiruvasagam and Srinivasan 2021).

The findings also raise difficulties for the partial awareness hypothesis (Kouider et al. 2010) and the LoP hypothesis (Windey et al. 2014, Windey and Cleeremans 2015, Jimenez et al. 2020). The partial awareness account attributes gradedness to different features reaching the global workspace at different times. Our data show that gradedness varies for the same feature (scene gist) as a function of load, implying that attention shapes gradedness within a single feature dimension. The LoP hypothesis treats gradedness as fixed for a given level of processing and varying across levels. Our finding that attentional load modulates gradedness within the level associated with gist perception suggests that LoP needs to incorporate attentional factors (Fazekas and Overgaard 2018) operating within a level, not only across them.

It should be noted that the present findings should not be taken to imply that consciousness is never all-or-none and is not a decisive test for discrete versus continuous theories of conscious perception. Classical psychophysical work has demonstrated that smooth psychometric functions can emerge under both continuous signal-detection frameworks and all-or-none detection models in which there is jitter at the trial level (Green and Swets 1966, Kingdom and Prins 2016). However, the focus in classical psychophysics has been primarily on psychometric functions for accuracy. Recent studies arguing for gradedness have focused primarily on PAS ratings functions and more specifically the slope parameter of the PAS ratings functions to investigate gradedness (Anzulewicz et al. 2015, Fazekas and Overgaard 2018, Jimenez et al. 2020, Overgaard et al. 2006, Sandberg et al. 2010, Windey et al. 2013, 2014). Our study also follows the same rationale for investigating gradedness of scene perception and indicating scene perception is probably graded with less gradedness when attentional resources are less to perform the task.

### Implications for the relationship between attention and awareness

The results also bear on the debate over whether attention is necessary for visual awareness (Cohen and Dennett 2011, Koch and Tsuchiya 2007, Maier and Tsuchiya 2021, Matthews et al. 2018, Pitts et al. 2018, Tsuchiya and Koch 2009, Van Boxtel et al. 2010). Theories arguing that attention is unnecessary have often cited gist perception as a case of awareness without top-down attention, based primarily on evidence that gist is not disrupted under dual task conditions (Li et al. 2002). Other studies have found the opposite, with gist reduced under dual task demands (Cohen et al. 2011, 2012, Mack and Clarke 2012). Our data aligned with this second set of findings: perceptual load in a concurrent task influenced both the threshold and slope of objective gist perception performance and also shaped the gradedness of awareness through changes in PAS function slope. This adds to evidence that attention affects not only accuracy but appearance (Mishra and Srinivasan 2017, Carrasco and Barbot 2019) and that different aspects of attention, such as scope, alter visual awareness (Baijal and Srinivasan 2009). While these results do not establish that attention is strictly necessary for awareness, they strengthen the case for theories that assign an important role to attention (Cohen et al. 2012) and weaken the case for attention-independent accounts (Koch and Tsuchiya 2007).

### Implications for gist perception

A central argument against the necessity of attention for awareness has rested on gist perception (Koch & Tsuchiya, 2007), with claims that gist can be processed without attention (Li et al. 2002, Tsuchiya and Koch 2009). This view has been built into models of visual search that treat gist as a parallel process free of selection bottlenecks (Wolfe et al. 1994, 2021), but it has been challenged by dual task studies showing that demanding primary tasks reduce scene perception (Cohen et al. 2011, 2012, Mack and Clarke 2012). Our findings add to these challenges. Manipulating perceptual load in a concurrent task affected scene identification and went further by showing that attention to a primary task influenced not only the accuracy but also the gradedness of scene perception.

### Limitations

The current study used indoor versus outdoor categorization to look at the effects of load on scene perception. Further studies would need to look at other aspects of scene perception. Multiple statistical parameters are supposed to underlie scene perception. The current study manipulated contrast or external noise but manipulating other important parameters that influence scene perception would allow us to form better theories of the effects of attention on scene perception.

## Conclusion

In the current study, we provide evidence for the modulation of gradedness of awareness of natural scenes by attention under different load conditions. Using a psychophysical approach across two experiments, we found that increased perceptual load in a primary task led to a more graded awareness of scene-gist in a secondary task, as indicated by changes in the slope of psychometric functions derived from PAS ratings. These findings challenge strictly all-or-none theories of consciousness and generally support theories that allow for gradedness of consciousness. The results also provide contrary evidence to the claim that gist perception occurs independently of attention. Experiment 2 results indicate that the mechanism underlying changes in gradedness due to load is external noise suppression. Taken together, these findings suggest that models of consciousness must account for flexible and context-dependent degrees of awareness shaped by different attentional processes and task demands. Future studies should further investigate how other forms of attention such as spatial or object-based attention influence gradedness of consciousness.

## Supplementary Material

Supplementary_Material_R2_for_proof_niag054

## Data Availability

Data summaries are available for access in the online repository *via* the link: https://osf.io/8fw4a/overview?view_only=39b9d773fd2842c4bfd9166243966708.

## Artificial intelligence

No AI assisted technologies were used in this research or creation of this article.
