# Supplementary material for: Gradedness of gist perception with scenes depends on concurrent task load: awareness under high load is more graded due to noise suppression
Source: Neurosci Conscious. 2026 Aug 24;2026(1):niag054. doi: 10.1093/nc/niag054 (PMC13631927; doi:10.1093/nc/niag054)
Supplement: Supplementary file 1 [file Supplementary_Material_R2_for_proof_niag054.docx]

**Supplementary Material**

**Sandberg et al. (2011) based analysis**

**Analysis 1: By Load-type analysis for each curve separately**

The nonlinear mixed modelling analysis was performed with load-type as a within subjects fixed factor and subjects as a random factor for all the 4 parameters of each curve-type separately (total 8 parameters). Experiment 1 results are presented in Tables S1 and S2 as well as Figure S1. Experiment 2 results are presented in Tables S3 and S4 as well as Figure S2.

**Experiment 1 results**

Table S1: Accuracy analysis results

| Parameter | Predictor | Difference | Std. Error | *t*-value | *p*-value |
| --- | --- | --- | --- | --- | --- |
| a | Low Load | -0. 0141 | 0.0194 | -0.7315 | 0.4649 |
| b | Low Load | -0.0076 | 0.0096 | -0.7938 | 0.4274 |
| c | Low Load | -0.0172 | 0.0033 | -5.1634 | < 0.0001 |
| d | Low Load | -0.0051 | 0.00274 | -1.8696 | 0.0623 |

**Intercept is the High Load condition*

Table S2: PAS ratings analysis results

| Parameter | Predictor | Difference | Std. Error | *t*-value | *p*-value |
| --- | --- | --- | --- | --- | --- |
| a | Low Load | 0.0823 | 0.0370 | 2.2469 | 0.0252 |
| b | Low Load | 0.0349 | 0.0111 | 3.1311 | 0.0019 |
| c | Low Load | 0.0027 | 0.0043 | 0.6393 | 0.5230 |
| d | Low Load | -0.0082 | 0.0032 | -2.5757 | 0.0104 |

Figure S1. (A) Average accuracy (objective task) and (B) Proportion of PAS ratings (subjective task) in high (red curve) and low load (blue curve) conditions at each contrast level of the scene-images in experiment 1.

**Experiment 2 results**

*Table S3: Accuracy analysis results*

| Parameter | Predictor | Value | Std. Error | *t*-value | *p*-value |
| --- | --- | --- | --- | --- | --- |
| a | Low Load | -0.0076 | 0.0081 | -0.9309 | 0.3523 |
| b | Low Load | 0.0296 | 0.0108 | 2.7245 | 0.0066 |
| c | Low Load | 10.8366 | 1.9455 | 5.5699 | < 0.001 |
| d | Low Load | -2.7010 | 1.7053 | -1.5838 | 0.1137 |

*Table S4: PAS rating analysis results*

| Parameter | Predictor | Value | Std. Error | *t*-value | *p*-value |
| --- | --- | --- | --- | --- | --- |
| a | Low Load | -0.0510 | 0.0198 | -2.5686 | 0.0104 |
| b | Low Load | 0.0686 | 0.0214 | 3.2009 | 0.0014 |
| c | Low Load | 2.7144 | 2.5784 | 1.0527 | 0.2929 |
| d | Low Load | -13.9409 | 2.5970 | -5.3680 | <0.001 |

Figure S2. (A) Average accuracy (objective task) and (B) Proportion of PAS ratings (subjective task) in high (red curve) and low load (blue curve) conditions at each noise level of the scene-images in experiment 2.

**Analysis 2: By Curve-type analysis for each load condition separately**

The nonlinear mixed-effects modelling analysis was performed with curve-type as a within subjects fixed factor and subjects and curve-type as a random factor. The analysis was performed for 3 parameters for each load type separately (total 6 parameters) in experiment 1 and for all the 4 parameters for each load type separately (total 8 parameters) in experiment 2.

**Experiment 1 results**

*Table S5: Low-load condition results*

| Parameter | Predictor | Difference | SE | *t*-value | *p*-value |
| --- | --- | --- | --- | --- | --- |
| b | Awareness | -0.0821 | 0.0121 | -6.7708 | < 0.0001 |
| c | Awareness | -1.8877 | 3.7693 | -0.5008 | 0.6168 |
| d | Awareness | 7.6321 | 2.4421 | 3.1251 | 0.0019 |

** Difference is with relative to values for the Accuracy curve*

** The lower asymptote was fixed for the model to converge.*

Unlike Sandberg et al (2011), the difference between the threshold parameter *c* for accuracy and awareness was not significantly different (Table S5). The result indicates that there is no statistical evidence for unconscious processing in the low load condition (Figure S3). Awareness is more graded than accuracy as in Sandberg et al (2011) in the low load condition.

*Table S6: High load condition results*

| Parameter | Predictor | Difference | SE | *t*-value | *p*-value |
| --- | --- | --- | --- | --- | --- |
| b | Awareness | -0.1096 | 0.0158 | -6.9237 | < 0.0001 |
| c | Awareness | -20.1130 | 5.4313 | -3.7031 | < 0.0001 |
| d | Awareness | 14.8062 | 3.6362 | 4.0718 | 0.0004 |

** Difference is with relative to values for the Accuracy curve*

** The lower asymptote was fixed for the model to converge.*

The difference between the threshold parameter *c* for accuracy and awareness was significantly different in the high load condition (Table S6). However, the threshold difference is negative indicating that awareness does not lag accuracy but leads in the high load condition (Figure S3). Awareness is more graded than accuracy as in Sandberg et al (2011) in the high load condition.

Figure S3. Accuracy (orange curve) and awareness (green curve) for each level of contrast of scene images for (A) low load, and (B) high load conditions in experiment 1.

**Experiment 2 results**

*Table S7: Low load analysis results*

| Parameter | Predictor | Value | Std. Error | *t*-value | *p*-value |
| --- | --- | --- | --- | --- | --- |
| a | Awareness | -0.0099 | 0.0099 | -1.0070 | 0.3143 |
| b | Awareness | -0.0024 | 0.0155 | -0.1592 | 0.8735 |
| c | Awareness | -13.5775 | 4.6171 | -2.9406 | 0.0034 |
| d | Awareness | 8.7299 | 1.7024 | 5.1279 | < 0.0001 |

The difference between the threshold parameter *c* for accuracy and awareness is significantly different (Table S7). However, the difference was negative indicating that awareness does not lag accuracy in the low load condition but leads in the low load condition (Figure S4). Awareness is more graded than accuracy as in Sandberg et al (2011) in the low load condition.

*Table S8: High load analysis results*

| Parameter | Predictor | Value | Std. Error | *t*-value | *p*-value |
| --- | --- | --- | --- | --- | --- |
| a | Awareness | -0.0009 | 0.0140 | -0.0061 | 0.9951 |
| b | Awareness | 0.0214 | 0.0183 | 1.1688 | 0.2430 |
| c | Awareness | -9.9844 | 4.6973 | -2.1255 | 0.0340 |
| d | Awareness | 13.8244 | 3.2852 | 4.2080 | <0.0001 |

The difference between the threshold parameter *c* for accuracy and awareness is significantly different (Table S8). However, the difference was negative indicating that awareness does not lag accuracy in the low load condition but leads in the low load condition (Figure S4). Awareness is more graded than accuracy as in Sandberg et al (2011) in the low load condition.

Figure S4. Figure S3. Accuracy (orange curve) and awareness (green curve) for each level of noise in scene images for (A) low load, and (B) high load conditions in experiment 2.
